# Supplementary material for: Emergence of ferroelectricity in Sn-based perovskite semiconductor films by iminazole molecular reconfiguration
Source: Nat Commun. 2025 Jan 3;16:365. doi: 10.1038/s41467-024-55113-0 (PMC11698826; doi:10.1038/s41467-024-55113-0)
Supplement: Supplementary file 1 — Supplementary Information [file 41467_2024_55113_MOESM1_ESM.pdf]

Supplementary Information

Emergence of ferroelectricity in Sn-based perovskite semiconductor films by iminazole molecular reconfiguration

Yu Liu[1,2], Shuzhang Yang[1], Lina Hua[1], Xiaomin Yang[1], Enlong Li[1], Jincheng Wen[1], Yanqiu Wu[1], Liping Zhu[3], Yingguo Yang[4], Yan Zhao[1], Zhenghua An[3], Junhao Chu[1,2], and Wenwu Li[1,2,]*

[1]State Key Laboratory of Photovoltaic Science and Technology, Institute of Optoelectronics, Department of Materials Science, Fudan University, Shanghai 200433, China.

[2]Shanghai Frontiers Science Research Base of Intelligent Optoelectronics and Perception, Fudan University, Shanghai 200433, China.

[3]State Key Laboratory of Surface Physics, Institute for Nanoelectronic Devices and Quantum Computing, Department of Physics, Fudan University, Shanghai 200433, China.

[4]School of Microelectronics, Fudan University, Shanghai 200433, China.

Email: liwenwu@fudan.edu.cn

Experimental details:

All the thin film materials were prepared in the nitrogen-filled glove box. All the devices were fabricated and measured in the nitrogen-filled glove box.

Chemicals: N,N-dimethylformamide (anhydrous, 99.8%, Adamas), dimethyl sulfoxide (anhydrous, 99.7%, Adamas), formamidinium iodide (FAI, ≥99.50%, Xi'an Yuri Solar Co., Ltd), phenethylammonium iodide (PEAI, ≥99.50%, Xi'an Yuri Solar Co., Ltd), caesium iodide (CsI, >99.90%, Xi'an Yuri Solar Co., Ltd), SnI_2 (anhydrous, beads, 99.99% trace-metal basis, Sigma-Aldrich), tin(ii) fluoride (99.00%, Sigma-Aldrich), 2-Methylbenzimidazole (MBI, 98%, Aladdin) and chlorobenzene (anhydrous, 99.80%, Sigma-Aldrich).

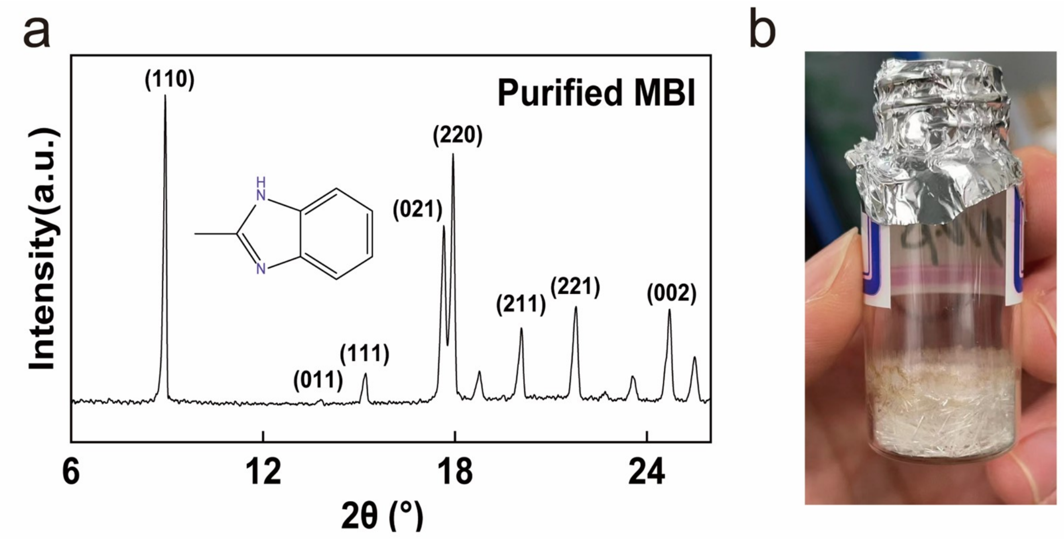

Supplementary Fig. 1: (a) the X-ray diffraction (XRD) pattern of purified MBI powders, the insert image shows the structural formula of MBI molecular; (b) the optical microscopy of the purified MBI powders.

To ensure the purity of the MBI molecules, we recrystallized the MBI powders (see Supplementary Fig.1b). The phase purity was confirmed by powder X-ray diffraction (PXRD) (see Supplementary Fig.1a)[1].

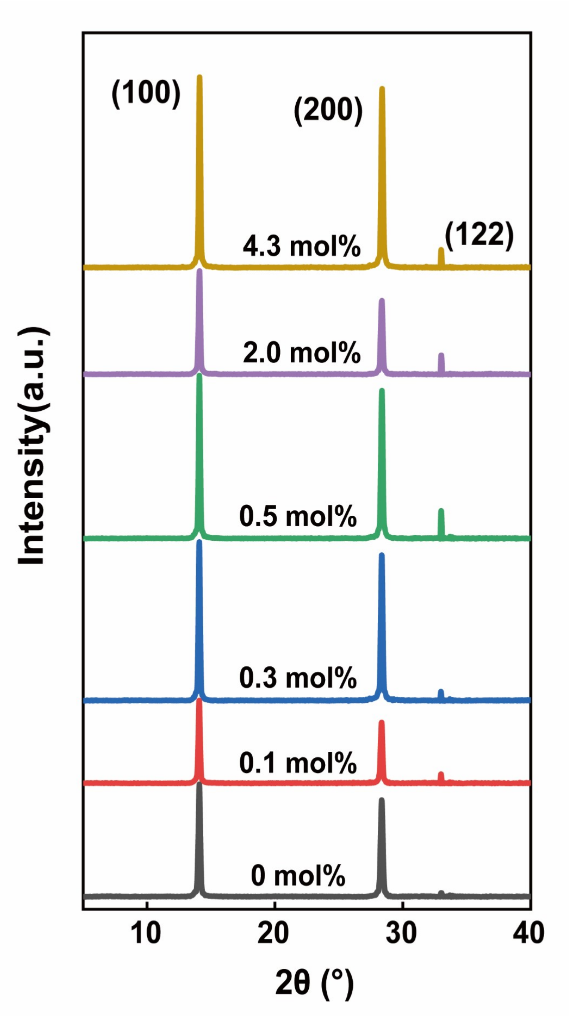

Supplementary Fig. 2: XRD patterns of the Sn-based perovskite films doping with different MBI concentrations.

Supplementary Table.1: Fullwidth at half-maximum (FWHM) of each crystal plane in XRD of perovskite films with different MBI concentrations.

MBI (mol%) \ FWHM	(100)
0	0.168
0.1	0.151
0.3	0.136
0.5	0.136
2.0	0.134
4.3	0.130

Supplementary Table.2: Peak position of (100) crystal plane in XRD of perovskite films with different MBI concentrations.

MBI (mol%) \ Peak position	(100)
0	14.08
0.1	14.08
0.3	14.08
0.5	14.08
2.0	14.10
4.3	14.10

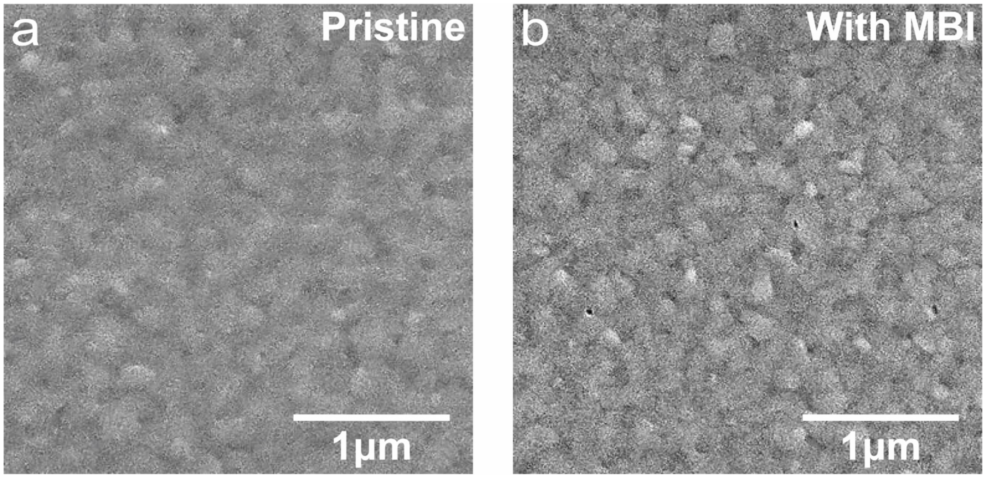

Supplementary Fig. 3: The SEM images of pristine and with MBI perovskite films.

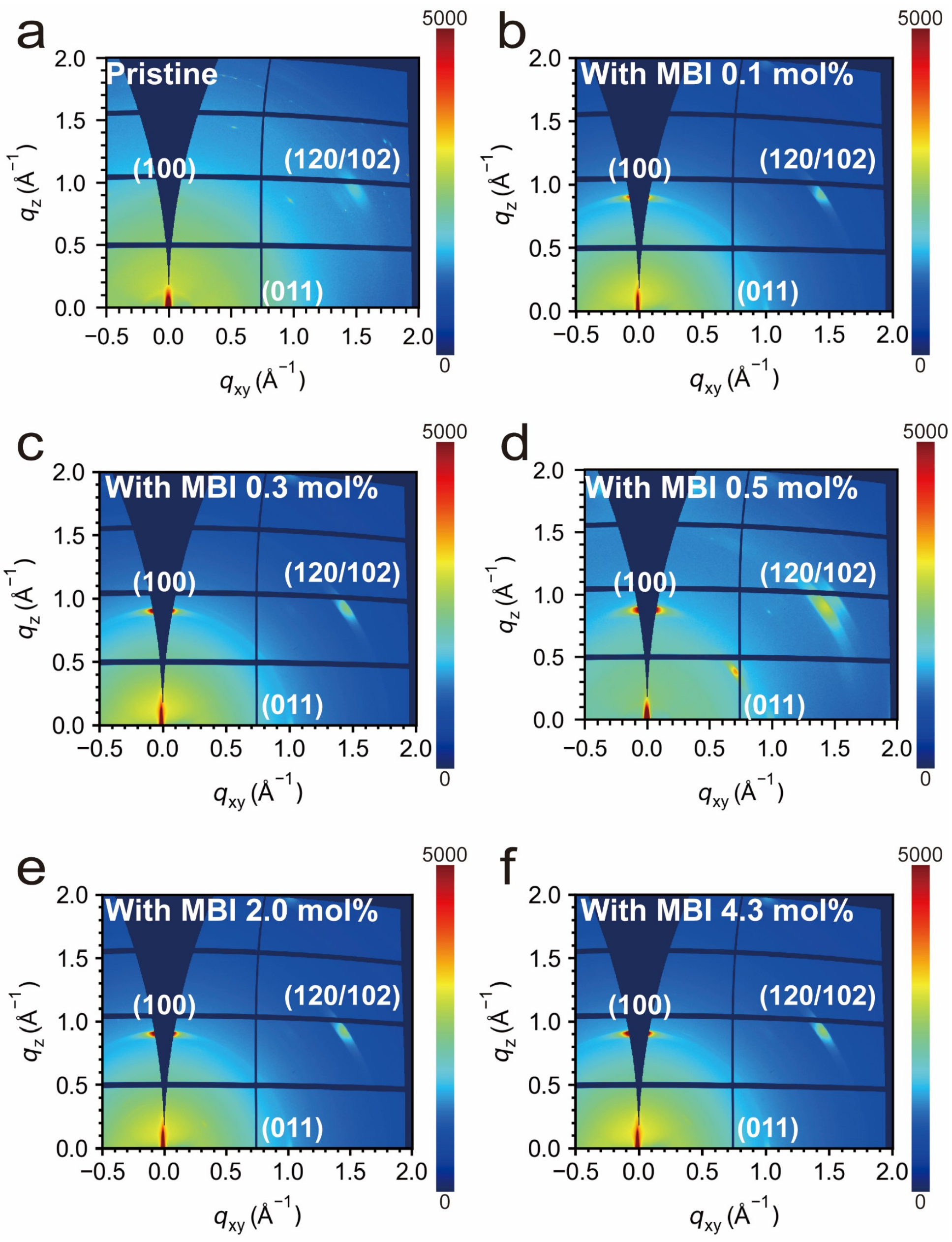

Supplementary Fig. 4: The GIWAXS data of Sn-based perovskite films with different doping concentrations of MBI molecule: (a) Pristine, (b) 0.1 mol%, (c) 0.3 mol%, (d) 0.5 mol%, (e) 2.0 mol%, (f) 4.3 mol%.

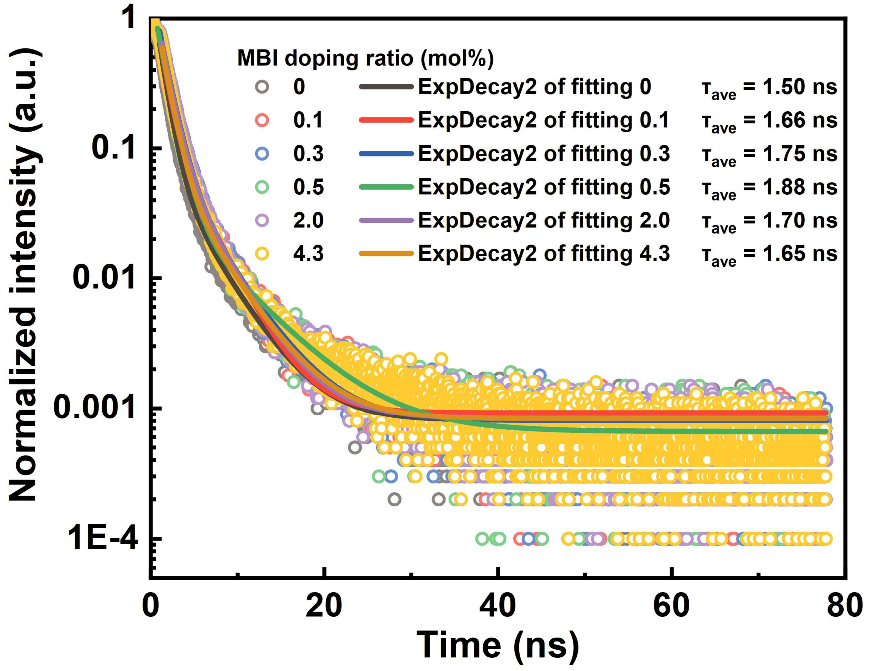

Supplementary Fig. 5: TRPL results of the Sn-based perovskite films with different doping concentrations of MBI molecule.

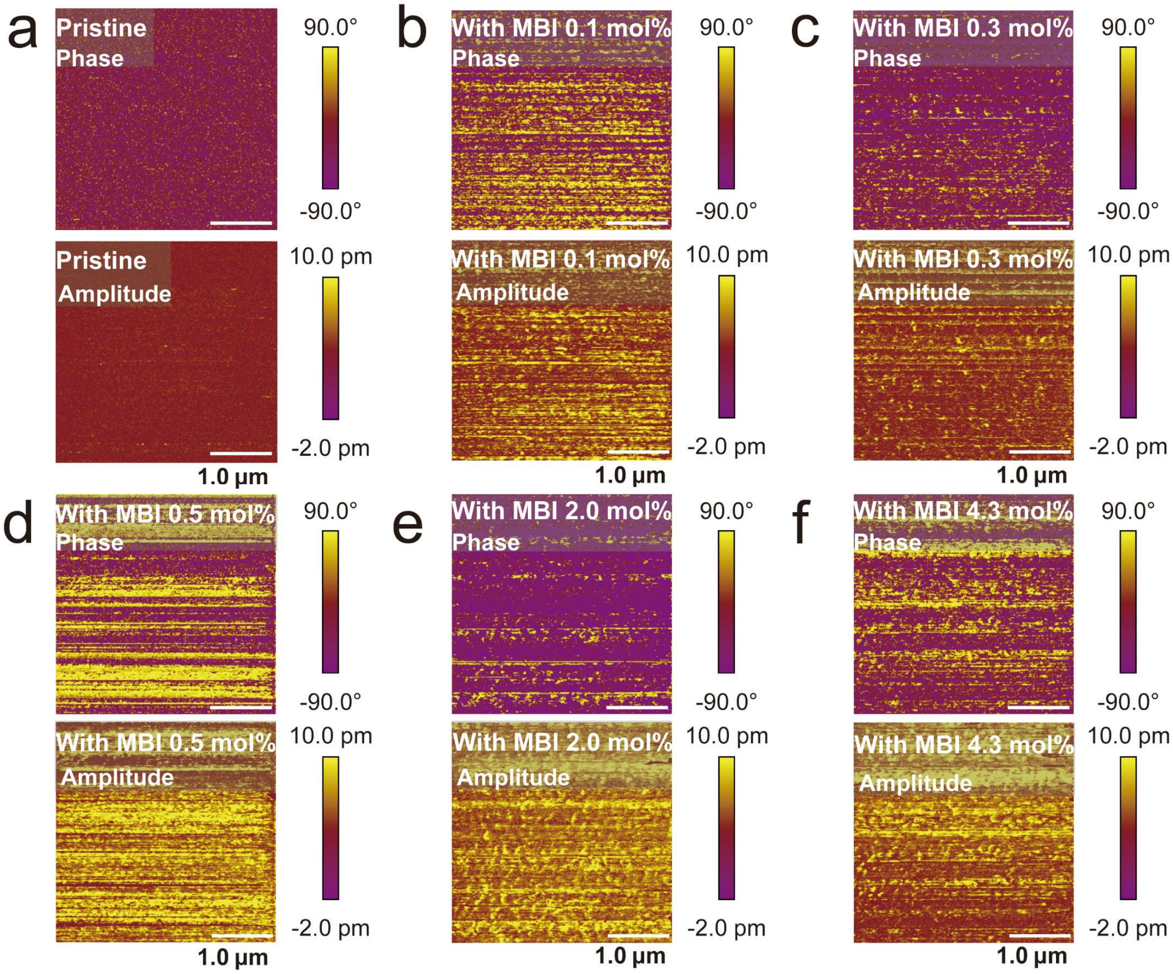

Supplementary Fig. 6: The piezoelectric atomic force microscope (PFM) phase and amplitude images of Sn-based perovskite films with different doping concentrations of MBI molecule: (a) Pristine, (b) 0.1 mol%, (c) 0.3 mol%, (d) 0.5 mol%, (e) 2.0 mol%, (f) 4.3 mol%.

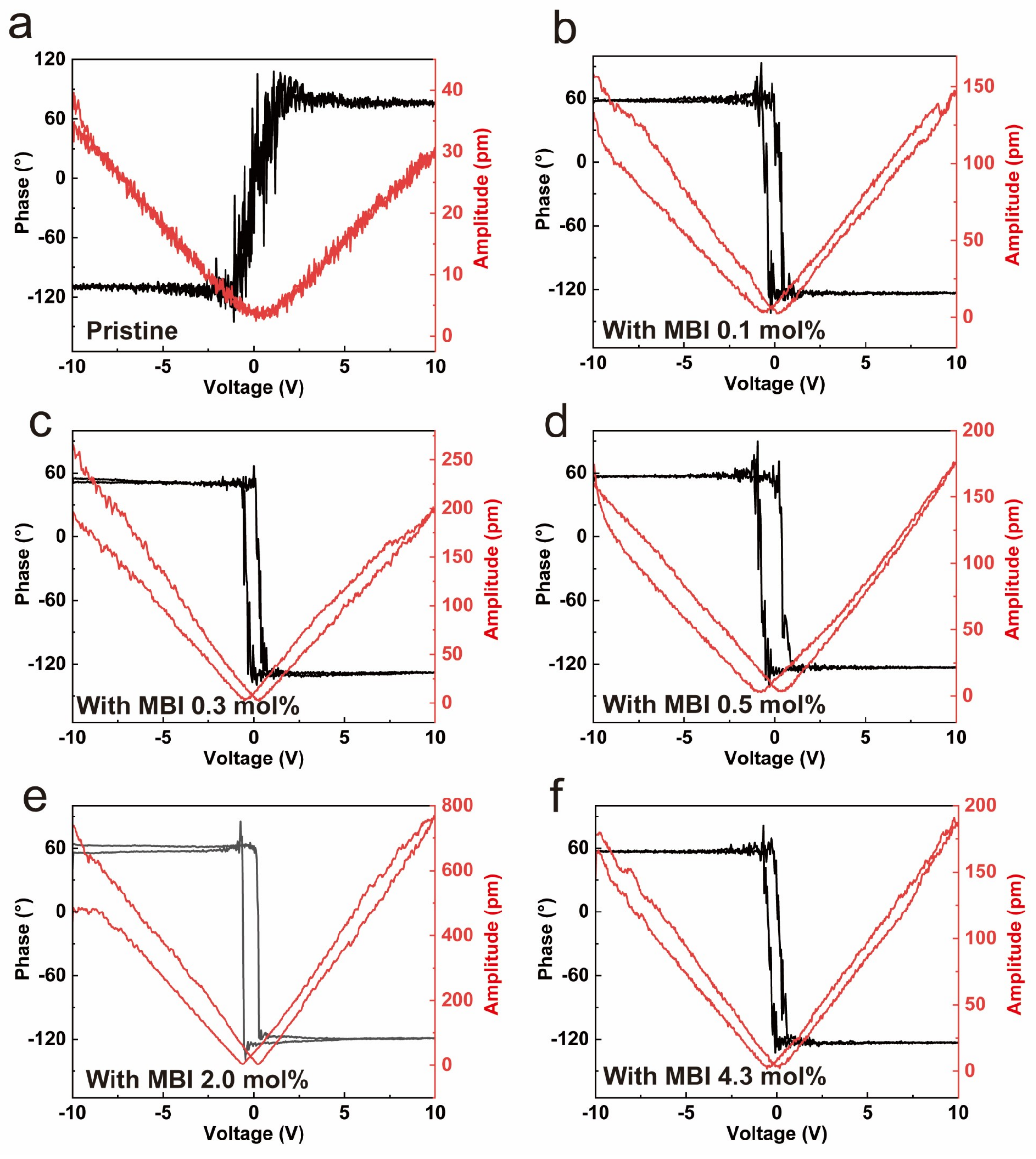

Supplementary Fig. 7: Local switching spectroscopy PFM (SS-PFM) phase and SS-PFM amplitude of Sn-based perovskite films with different doping concentrations of MBI molecule: (a) Pristine, (b) 0.1 mol%, (c) 0.3 mol%, (d) 0.5 mol%, (e) 2.0 mol%, (f) 4.3 mol%.

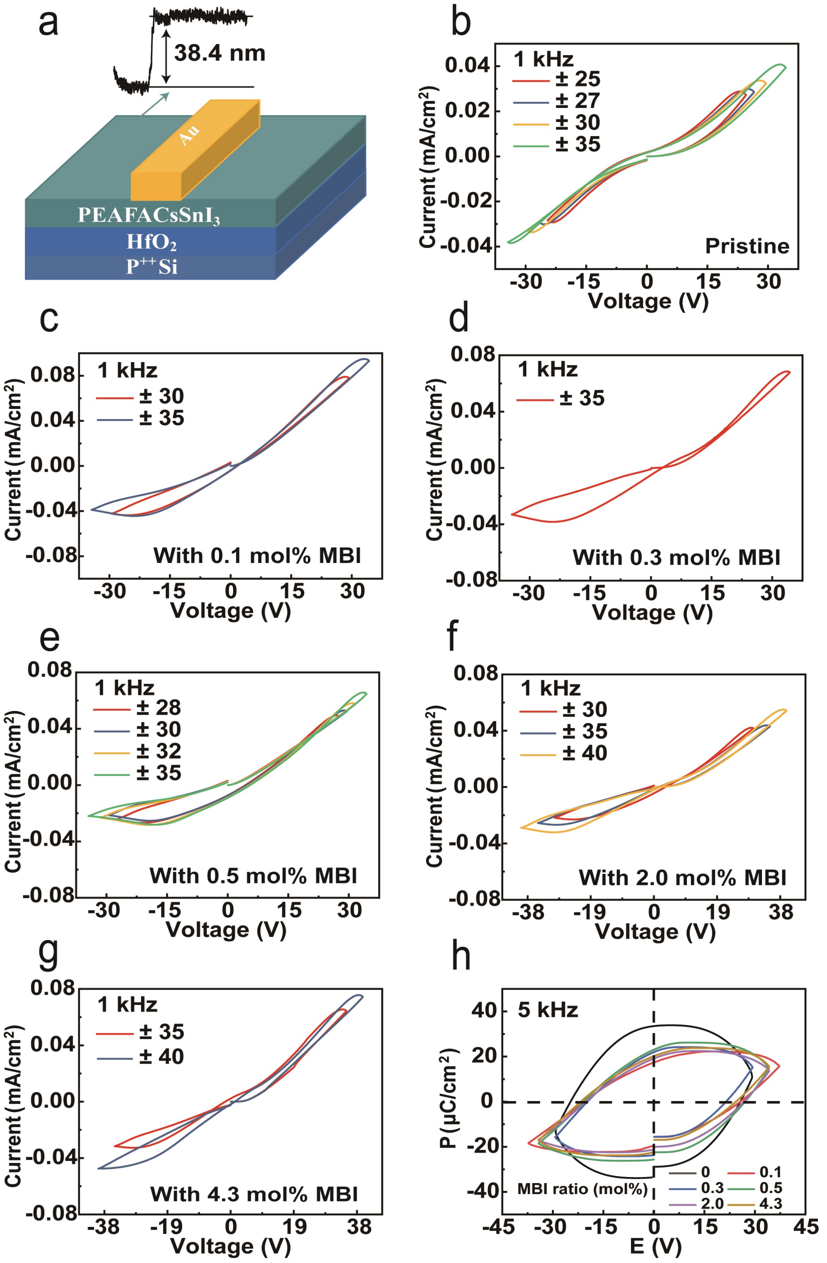

Supplementary Fig. 8 (a) the MISM structure diagram to measure the ferroelectricy of Sn-based perovskite films. Inset shows the thickness (about 38 nm) of the perovskite films; (b)-(g) I-V test results of ferroelectric analysis tester of the Sn-based perovskite films with different doping concentrations of MBI molecule; (h) P-V test results of ferroelectric analysis tester of the Sn-based perovskite films with different doping concentrations of MBI molecule.

Due to the semiconductor nature of the tin-based perovskite film, it exhibits a substantial leakage current, making it challenging to obtain its polarization reversal current. In this work, the metal-

insulator-semiconductor-metal (MISM) structure was employed for testing, as depicted in Supplementary Figure 8a. This configuration can effectively reduce the leakage current, thereby facilitating the observation of the polarization reversal current in the ferroelectric semiconductor film materials. The absence of polarization reversal current in the positive direction is due to the inconsistent contact between the upper and lower interfaces of the semiconductor[2,3]. The upper interface, which contacts the conductor, experiences a leakage current that obscures the polarization reversal current. In contrast, the lower interface, in contact with the insulator, effectively blocks leakage current, thereby allowing the polarization reversal current to be observed. The pristine I-V curve (Supplementary Fig. 8b) does not exhibit a reversal peak in the polarization current, and the pristine P-V curve (Supplementary Fig. 8h) presents an annular shape, indicating that the perovskite film is not ferroelectric.

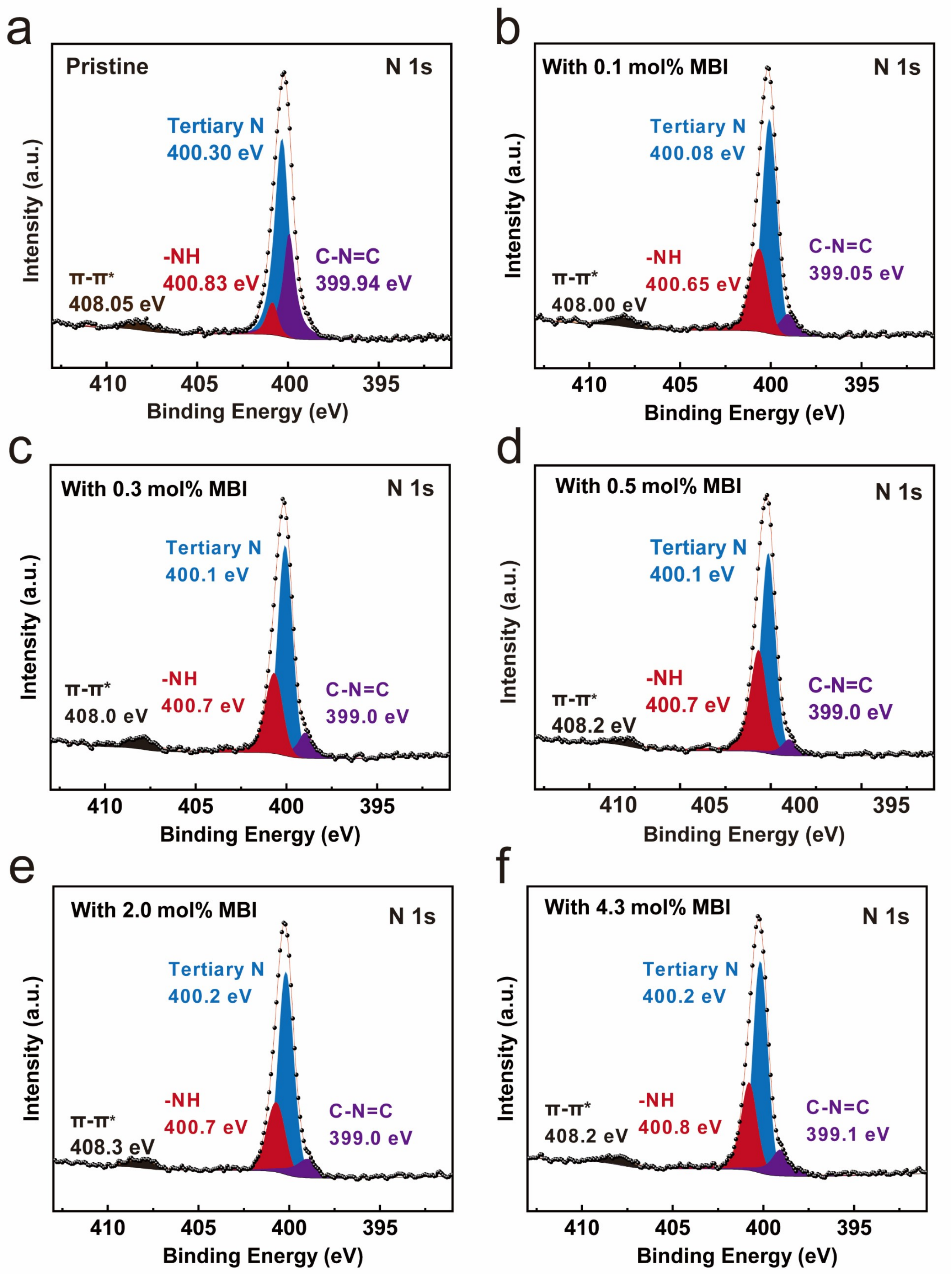

Supplementary Fig. 9: The X-ray photoelectron spectroscopy (XPS) (N 1s) of Sn-based perovskite films with different doping concentrations of MBI molecule: (a) Pristine, (b) 0.1 mol%, (c) 0.3 mol%, (d) 0.5 mol%, (e) 2.0 mol%, (f) 4.3 mol%.

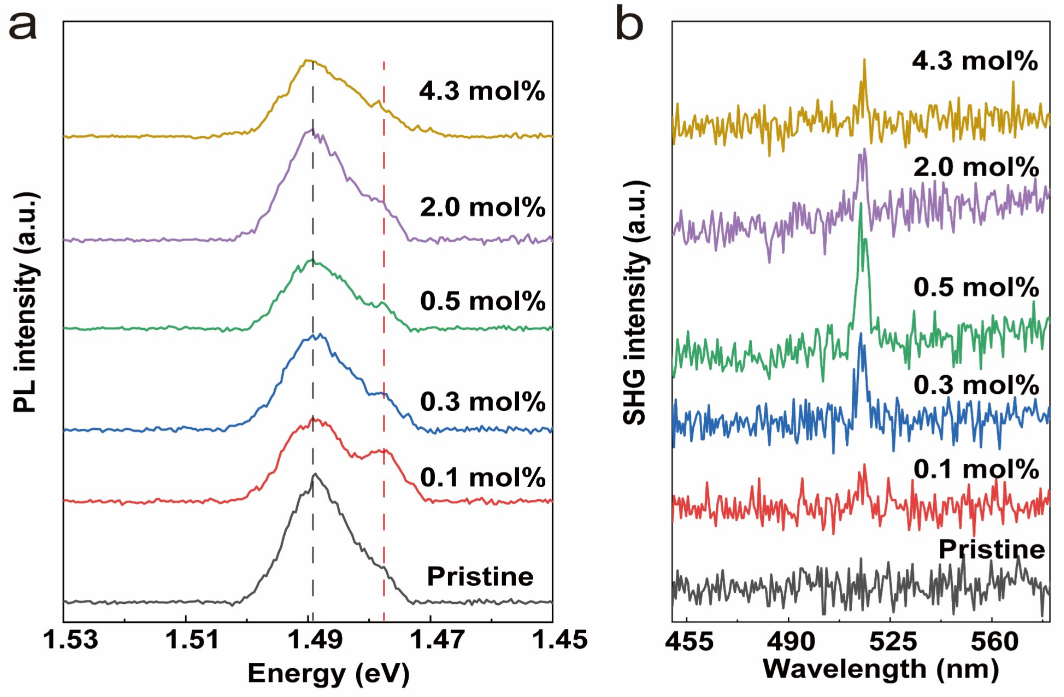

Supplementary Fig. 10: (a) photoluminescence spectroscopy and (b) second harmonic generation (SHG) spectra of the Sn-based perovskite films with different doping concentrations of MBI molecule.

After the introduction of MBI, the energy levels of the Sn-based perovskite undergo splitting, as evidenced by the emergence of a shoulder peak in the photoluminescence (PL) spectrum. The broad absorption band of the perovskite contributes to a reabsorption effect, where photons emitted from shallower energy states are reabsorbed by deeper states[4]. As a result, while the overall crystallinity remains high as confirmed by XRD measurements, the PL intensity does not show a corresponding enhancement.

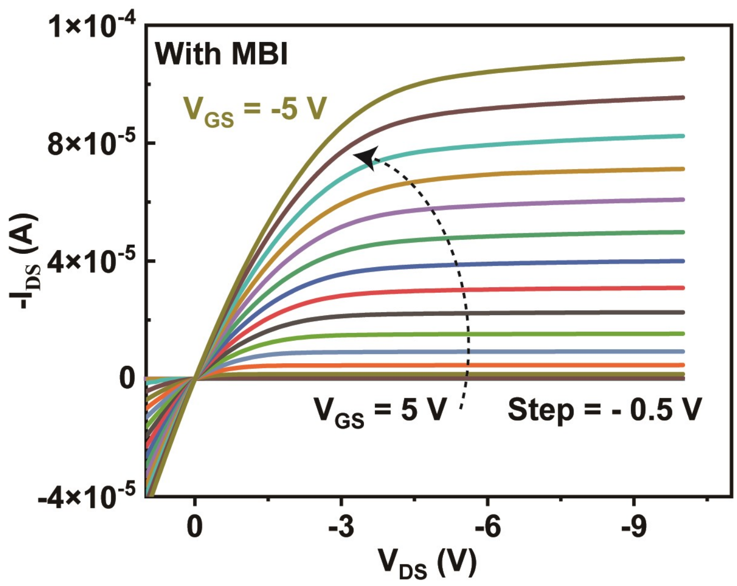

Supplementary Fig. 11: The output curves of the Sn-based perovskite ferroelectric transistor, doping with 0.5 mol% MBI molecule.

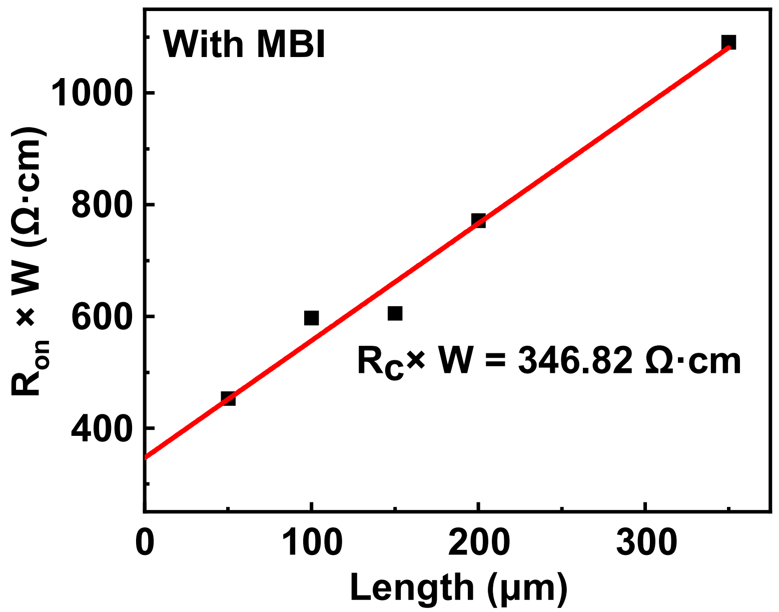

Supplementary Fig. 12: Extraction of with MBI perovskite transistor contact resistance by TLM method.

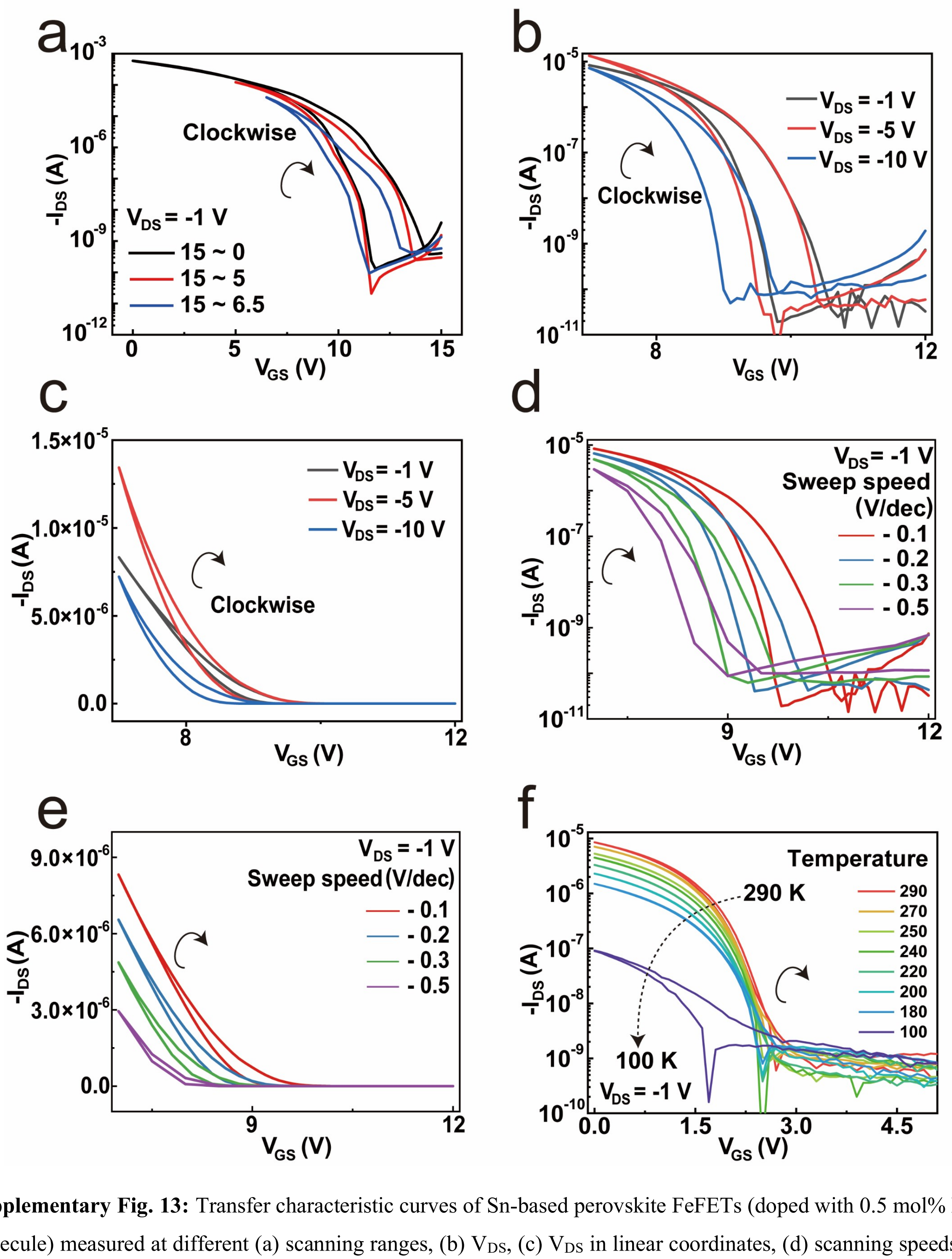

Supplementary Fig. 13: Transfer characteristic curves of Sn-based perovskite FeFETs (doped with 0.5 mol% MBI molecule) measured at different (a) scanning ranges, (b) V_{DS}, (c) V_{DS} in linear coordinates, (d) scanning speeds, (e) scanning speeds in linear coordinates, and (f) temperatures.

The ion migration can influence the size and direction of the hysteresis window in the transfer characteristic curve. Since the speed of ion migration is constant under a given electric field, a

change in the size of the hysteresis window with varying scanning speeds would indicate the presence of ion migration. Conversely, a lack of change suggests an absence of ion migration. Additionally, ion migration is significantly inhibited at low temperatures. Therefore, the transfer characteristic curve at low temperatures can more accurately reflect whether the hysteresis window is due to ferroelectric hysteresis. Across different scanning ranges and V_{DS}, the transfer characteristic curve consistently shows that the backward current exceeds the forward current (Supplementary Fig.13a, 13b, and 13c). At various scanning speeds, the size of the hysteresis window remains largely unchanged at approximately 0.5 V. In both linear and logarithmic coordinates, the backward current is always greater than the forward current (Supplementary Fig.13d and 13e).

The hysteresis observed in the transfer curve of ferroelectric perovskite transistors primarily arises from defect trapping and ferroelectric polarization. The extent of defect trapping is temperature-dependent[5]; at lower temperatures, the thermal activation energy is insufficient for carriers to escape from defect sites after being captured and certain defect states may become "frozen", meaning that their ability to interact with carriers (i.e., to capture or release them) is diminished[6]. This is often observed in semiconductors where deep-level defects, which require significant energy for interaction, become inactive at low temperatures[7]. Consequently, the impact of defect capture and release diminishes as temperature decreases. In contrast, the effect of ferroelectric polarization remains largely independent of temperature[8]. Therefore, at low temperatures, the influence of the defect field is reduced compared to that at room temperature, allowing the ferroelectric polarization effect to become more prominent.

The minimum voltage presented in Fig. 5b is 4V, whereas in Supplementary Fig. 13f, it decreases to 0V. Hysteresis predominantly manifests in the subthreshold region of the transfer curve. However, as the scanning range expands and reaches the saturation region, the degree of hysteresis decreases. With the voltage nearing 0 V, the increasing carrier concentration in the perovskite layer begins to shield the ferroelectric field. The saturation region with high carrier concentration is more sensitive to charge capture and release processes, while the subthreshold region has a smaller carrier concentration, resulting in a greater impact of defect capture and release on the transfer curve in the saturation region.

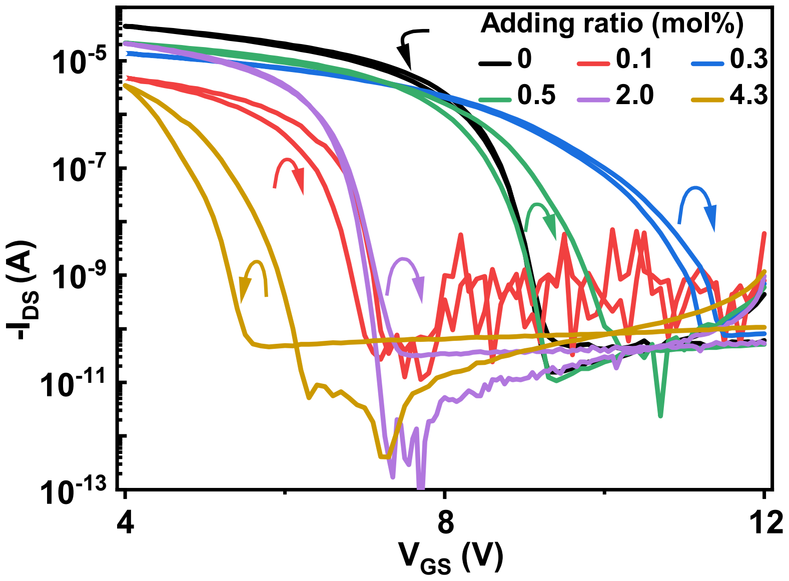

Supplementary Fig. 14: The transfer characteristic curves of the Sn-based perovskite FETs with different doping concentrations of MBI molecule.

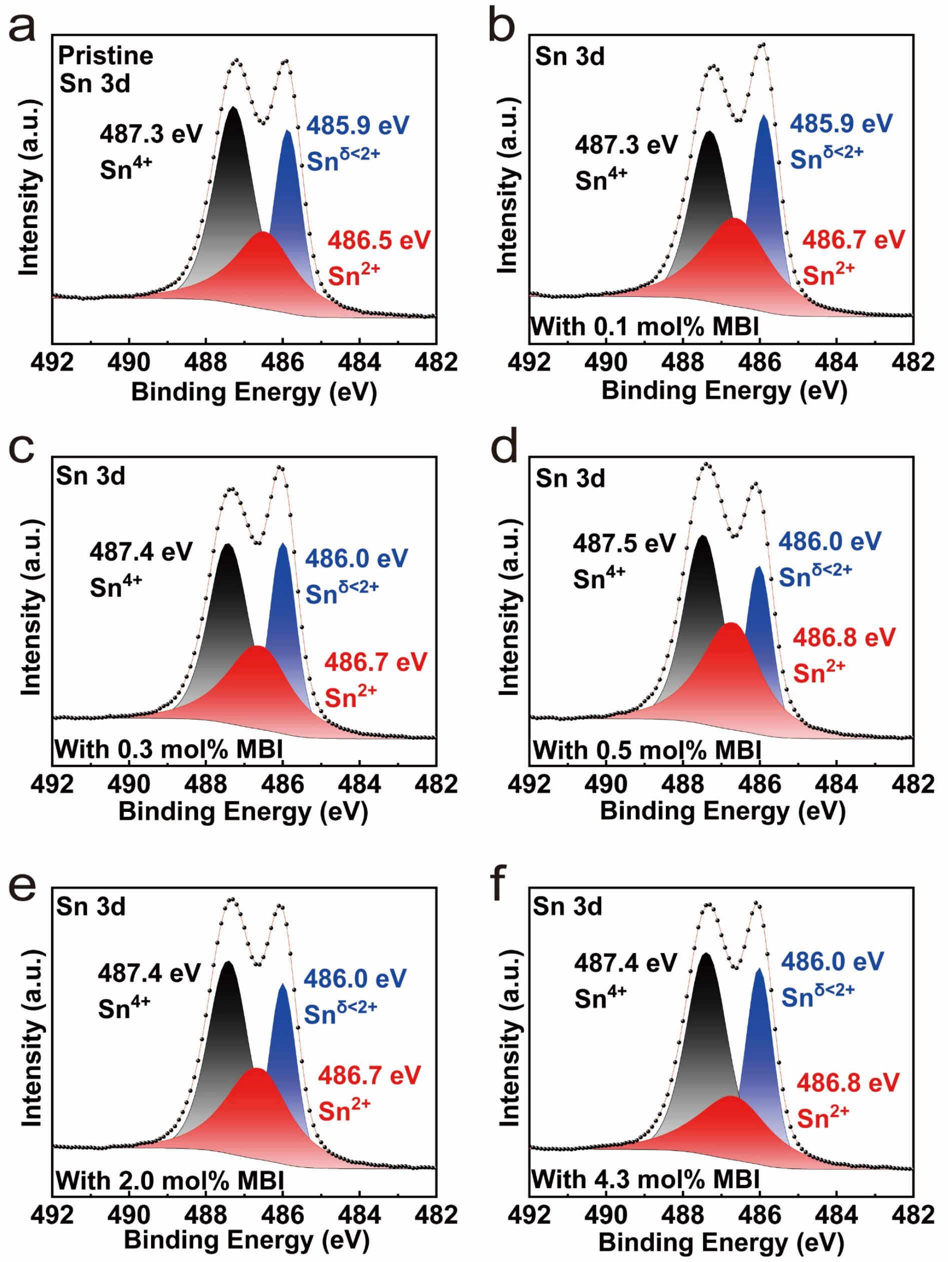

Supplementary Fig. 15: The X-ray photoelectron spectroscopy (XPS) (Sn 3d) of Sn-based perovskite films with different doping concentrations of MBI molecule: (a) Pristine, (b) 0.1 mol%, (c) 0.3 mol%, (d) 0.5 mol%, (e) 2.0 mol%, (f) 4.3 mol%.

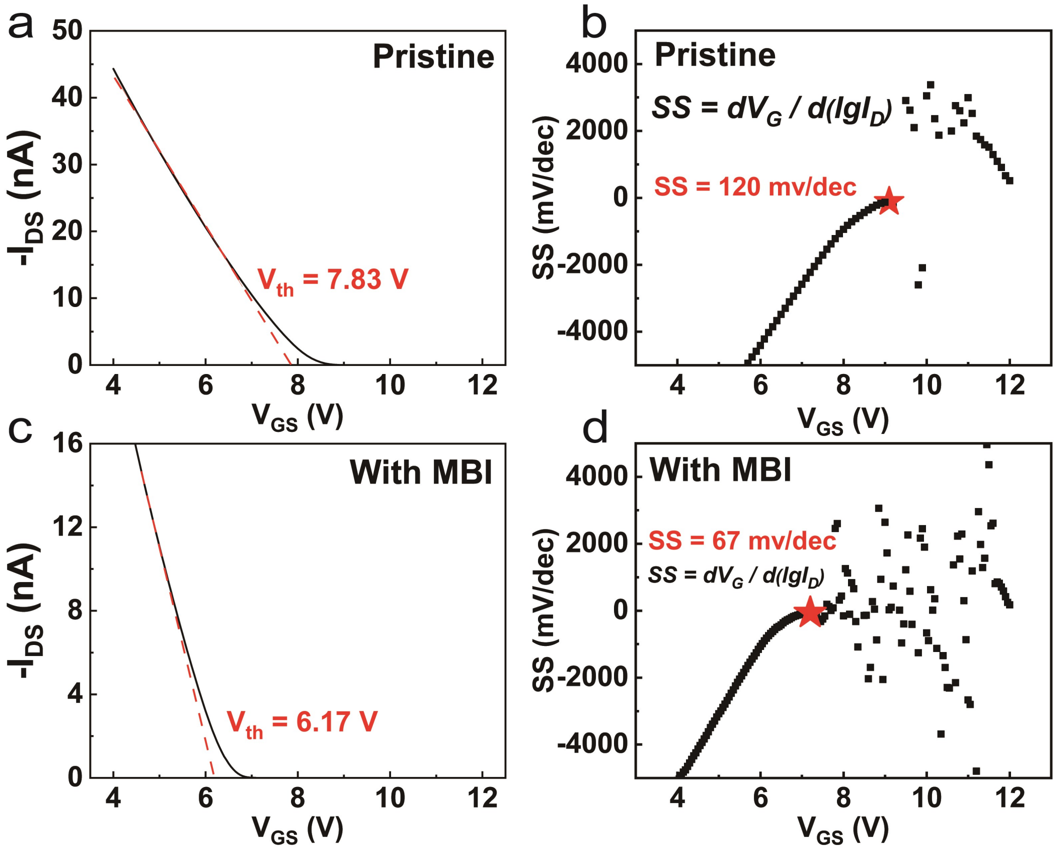

Supplementary Fig. 16: V_{th} and SS extraction methods for both pristine and MBI-doped perovskite transistors.

References

1. Balashova, E., Levin, A. A., Fokin, A., Redkov, A. & Krichevtsov, B. Structural properties and dielectric hysteresis of molecular organic ferroelectric grown from different solvents. *Crystals.* **11**, 1278 (2021).

2. Xie, X. *et al.* Effects of excess Bi on structure and electrical properties of $BiFeO_3$ thin films deposited on indium tin oxide substrate using sol–gel method. *J Mater Sci: Mater Electron* **26**, 10095–10101 (2015).

3. Maruno, S. *et al.* Model of leakage characteristics of $(Ba, Sr)TiO_3$ thin films. *Appl. Phys. Lett.* **73**, 954–956 (1998).

4. Gan Z., Xu H., Fu Y. Photon reabsorption and nonradiative energy-transfer-induced quenching of blue photoluminescence from aggregated graphene quantum dots. *J. Phys. Chem. C* **120**, 29432-29438 (2016).

5. Illarionov, Y. Y. et al. Long-term stability and reliability of black phosphorus field-effect transistors. *ACS Nano* **10**, 9543–9549 (2016).

6. Crowell, C. R. The Richardson constant for thermionic emission in Schottky barrier diodes. *Solid-State Electronics* **8**, 395–399 (1965).

7. Landi, G. et al. Correlation between electronic defect states distribution and device performance of perovskite solar cells. *Advanced Science* **4**, 1700183 (2017).

8. Liu, X. et al. Charge–ferroelectric transition in ultrathin $Na_{0.5}Bi_{4.5}Ti_4O_{15}$ flakes probed via a dual-gated full van der waals transistor. *Advanced Materials* **32**, 2004813 (2020).
